# Improving Adult Inpatient Discharge Planning and 72-Hour Follow-Up: A Regional Re-Audit Across Three Mental Health Sites

**DOI:** 10.1192/bjo.2026.11663

**Published:** 2026-06-30

**Authors:** Tajnin Mitu, Asmaa Elsayed, Anita Pierce, Mostafa Negm, Ishraq Elahi

**Affiliations:** 1 Betsi Cadwaldr University Health Board, Wrexham, United Kingdom; 2 Betsi Cadwaladr University Health Board, Wrexham, United Kingdom

## Abstract

**Aims:**

The period immediately following discharge from psychiatric inpatient mental health care is associated with heightened clinical vulnerability, including increased risk of relapse, crisis presentation and suicide. National standards from the National Confidential Inquiry into Suicide and Safety in Mental Health, NHS CQUIN targets and the Welsh Government Crisis Care Concordat recommend that all adult patients receive follow-up by a mental health professional within 72 hours of discharge and that discharge documentation comprehensively records risk, mental state, medication and care planning. A previous regional audit identified variable compliance and incomplete documentation across sites. This re-audit aimed to assess current adherence to these standards, evaluate whether implemented quality improvement measures had improved practice, and identify ongoing gaps to inform further service development.

**Methods:**

A retrospective clinical re-audit was undertaken across three adult inpatient units (East, Central and West) within Betsi Cadwaladr University Health Board. All eligible discharges occurring during June–July 2025 were included. Electronic discharge summaries and 72-hour follow-up records were reviewed using predefined criteria. Standards assessed included completion of follow-up within 72 hours and documentation of risk assessment, mental state examination (MSE), medication information, and a comprehensive discharge plan, alongside the quality of follow-up notes. Cases recorded as not applicable or refused were excluded. Missing or undocumented items were classified as non-compliant. Compliance rates were calculated overall and by site and compared with baseline findings from the initial audit.

**Results:**

Thirty-eight discharges were reviewed. Overall compliance with 72-hour follow-up was 78.4% (29/37). Documentation of comprehensive discharge plans was highest (86.5%), followed by MSE (78.8%) and risk assessment (72.7%). Medication documentation remained comparatively low at 63.6%. Considerable inter-site variation was observed. The Central site achieved 100% follow-up compliance and consistently strong documentation across domains. In contrast, the East site demonstrated lower follow-up completion (57%) and particularly poor medication documentation (20%), while the West site showed moderate follow-up rates (66.7%) with weaker recording of risk (46.2%) and medication (53.8%). Compared with baseline data, overall follow-up and discharge planning showed modest improvement, but reliability of documentation remained inconsistent.

**Conclusion:**

Although small gains were demonstrated following initial interventions, substantial variation persists between sites, indicating inconsistent processes and handover practices. Standardised electronic templates, discharge checklists, automated alerts and routine audit feedback have been introduced to improve reliability. Ongoing quality improvement and targeted site support are required to achieve consistent adherence to national standards and to enhance safety during the high-risk transition from inpatient to community care.

Other authors-Dr. Aanika Nawer Hoque,Dr Nur Efina Mokhtar,Dr Charlotte Hague-Roberts

